# A Multichannel 2D Convolutional Neural Network Model for Task-Evoked fMRI Data Classification

**DOI:** 10.1155/2019/5065214

**Published:** 2019-12-31

**Authors:** Jinlong Hu, Yuezhen Kuang, Bin Liao, Lijie Cao, Shoubin Dong, Ping Li

**Affiliations:** ^1^School of Computer Science and Engineering, South China University of Technology, Guangzhou, China; ^2^College of Mathematics and Informatics, South China Agricultural University, Guangzhou, China; ^3^Faculty of Humanities, The Hong Kong Polytechnic University, Hong Kong, China

## Abstract

Deep learning models have been successfully applied to the analysis of various functional MRI data. Convolutional neural networks (CNN), a class of deep neural networks, have been found to excel at extracting local meaningful features based on their shared-weights architecture and space invariance characteristics. In this study, we propose M2D CNN, a novel multichannel 2D CNN model, to classify 3D fMRI data. The model uses sliced 2D fMRI data as input and integrates multichannel information learned from 2D CNN networks. We experimentally compared the proposed M2D CNN against several widely used models including SVM, 1D CNN, 2D CNN, 3D CNN, and 3D separable CNN with respect to their performance in classifying task-based fMRI data. We tested M2D CNN against six models as benchmarks to classify a large number of time-series whole-brain imaging data based on a motor task in the Human Connectome Project (HCP). The results of our experiments demonstrate the following: (i) convolution operations in the CNN models are advantageous for high-dimensional whole-brain imaging data classification, as all CNN models outperform SVM; (ii) 3D CNN models achieve higher accuracy than 2D CNN and 1D CNN model, but 3D CNN models are computationally costly as any extra dimension is added in the input; (iii) the M2D CNN model proposed in this study achieves the highest accuracy and alleviates data overfitting given its smaller number of parameters as compared with 3D CNN.

## 1. Introduction

Task-evoked functional Magnetic Resonance Imaging (fMRI) is the most common type of fMRI data in the study of brain functions based on the changing levels of blood oxygenation-level dependent (BOLD) signals. In task-evoked fMRI scanning, participants receive different task stimulation and simultaneously perform specific responses that lead to different BOLD signals, producing time series of three-dimensional volume of brain at millimetric spatial resolution within a task block.

A goal to use computational methods to classify task-evoked fMRI data is to potentially develop predictive models or systems that can recognize how the brain responds to different task stimulation [1]. The implications of these models are profound in terms of identifying the relationships among brain response, individual behavior, and cognitive task (i.e., the brain-behavior-cognition relationships).

Deep learning models have been successfully applied to the analysis of various fMRI data [2], such as convolutional neural networks (CNN), a class of deep neural networks, for their ability to extract local meaningful features which are shared in the entire dataset, due to CNN's shared-weights architecture and space invariance characteristics [3]. Compared to resting-state fMRI, task-evoked fMRI data involve the participant's response within a shorter time span, which can be represented as three-dimensional data covering the coronal, sagittal, and axial axes/planes of the brain [2].

In this paper, we focus on CNN models for classifying 3D voxel-wise fMRI data, especially task-evoked fMRI data. Currently, 2D and 3D CNN methods have been used to classify task-evoked voxel-wise fMRI data in the literature within two broad clusters. (i) The first cluster is 2D CNN models for fMRI data classification. Nathawani et al. [4] transformed 3D brain image into 2D mean-value image by computing mean values along the *z*-axis and trained 2D CNN for classifying fMRI data from word reading tasks. Hon and Khan [5] transformed each 3D brain image into 2D slices and calculated the image entropy of each slice and then extracted slices with the highest entropy for training the 2D CNN network. Zafar et al. [6] changed 3D brain image into multilayer 2D image as input to 2D CNN for feature extraction and selected the features by *t*-test to classify visual tasks with the SVM algorithm. (ii) The second cluster is 3D CNN models for fMRI data classification. Li et al. [7] used 3D CNN to learn informative features from whole brain for brain age prediction. Li et al. [8] computed the mean 3D image and the standard deviation 3D image for all voxels' time series in each sliding window and generated the 2-channel input 3D images for training their 2-channel 3D CNN model. Wang et al. [9] used 3D CNN to classify 4D task fMRI time series by regarding them as multichannel 3D input. In addition to models applied to the task-evoked fMRI data as discussed, CNN models have also been used for resting-state fMRI data classification; for example, Sarraf and Tofighi [10] used 2D CNN architecture LeNet-5 for Alzheimer's disease classification by a stack of 2D images converted from fMRI 4D data.

However, in many cases, it is challenging to compare different types of CNN models and design (or select) an optimal model for 3D fMRI data classification. Compared with 2D convolution, 3D convolution could extract the 3D spatial information from 3D fMRI data, but the demands for computational resources are high, and the number of model parameters increases exponentially as an extra dimension is added. Therefore, training of a 3D CNN model is computationally costly, and too many parameters can also cause data overfitting in supervised learning. Meanwhile, models with separable convolutions have been developed in computer vision research [11] to make computation more tractable. Specifically, the 3D separable convolutional neural networks (3D SepConv) were used in the context of structural MRI imaging for improving efficiency of regular 3D CNN [12], but the learning effectiveness of 3D SepConv model might be affected by less parameters in a convolution kernel as compared with general 3D CNN model, and 3D SepConv model also has more parameters than 2D CNN model. In contrast, 2D CNN models require less computational resource and fewer parameters, but they lose some spatial information of 3D fMRI data while gaining higher training efficiency.

To address this challenge, we study different types of CNN models for 3D fMRI data classification and propose M2D CNN, a novel multichannel 2D CNN model for the classification of the 3D fMRI data. The proposed model includes two stages:

- Transforming 3D fMRI images into multichannel 2D images for learning with multichannel 2D CNN network: first, we slice 3D fMRI images into a group of 2D fMRI images along with one dimension, where one sliced 2D fMRI image would be viewed as one channel image. The 2D CNN model would receive the multichannel 2D images as input, taking into account the images in different channels, such as RGB image [13]. In the MRI literature, 3 anatomical planes of brain are conventionally used by imaging researchers to describe brain images: coronal as *x*-coordinate, sagittal as *y*-coordinate, and axial as *z*-coordinate. We use three multichannel 2D CNN networks to learn and extract features of these planes, respectively.
- Integrating multichannel information from three 2D CNNs: to integrate the information from three multichannel 2D CNNs learning networks, we use a fully connected neural network to learn the three-dimensional information.

The proposed M2D CNN model thus uses 2D CNNs to handle the 3D brain images, which balances the learning ability and parameter-efficiency (fewer parameters) of the model. We examined model classification performance (accuracy, precision, and F1-score), model training time, number of parameters, training, and validation loss and compared our model against six other models as benchmarks in the classification of a large number of time-series whole-brain imaging data from a motor task in the Human Connectome Project (HCP).

## 2. Method

### 2.1. M2D CNN Model Architecture

**Figure 1:**
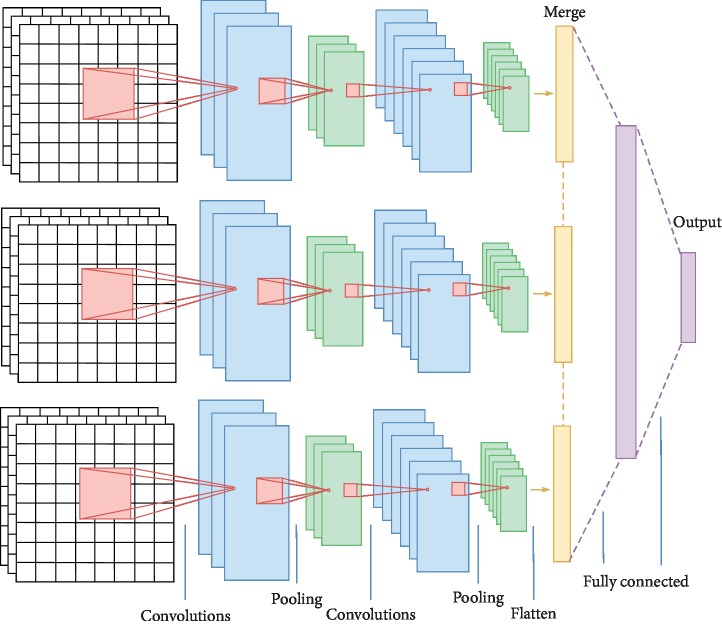
Architecture of the M2D CNN model.

As mentioned above, brain images are viewed along 3 anatomical planes, coronal as *x*-coordinate, sagittal as *y*-coordinate, and axial as *z*-coordinate. We use three 2D CNNs to extract features of these planes, respectively, and then combine three 2D CNN architectures in parallel, that is, the multichannel 2D CNN (M2D CNN). M2D CNN model consists of three parts of the 2D CNN architecture in parallel, plus a fully connected hidden layer to integrate the multichannel information. Each 2D CNN uses one type of multichannel 2D images as inputs and carries out the convolution computing independently. The outputs of three 2D CNN parts are flattened and concatenated into 1D features in series, which are input to the fully connected neural network for further learning. Finally, M2D CNN outputs the classification result. Since the concatenated features contain the features extracted from three orthogonal planes, M2D CNN takes into account the 3D spatial information. The architecture of the M2D CNN model is shown in Figure 1.

The M2D CNN architecture in this model consists of 3 input layers, six convolutional layers, six pooling layers, one merge layer, one fully connected layer, and one output layer. This use of multichannel 2D convolution can effectively improve the calculation compared to 3D convolution. The model details are as follows.

#### 2.1.1. Input Layer

Transform each fMRI 3D brain image into three multichannel 2D images. For a 3D brain image sample with the size of dim*X*  × dim*Y*  × dim*Z* (e.g., 3 × 3 × 3 mm), slice along the *x*-axis in per unit length to get dim*X* 2D images with the size of dim*Y*  × dim*Z* on coronal plane. Similarly, slice along the *y*-axis and the *z*-axis to get dim*Y* 2D images with the size of dim*X*  × dim*Z* on sagittal plane and dim*Z* 2D images with the size of dim*X*  × dim*Y* on axial plane. In this way, three groups of images based on different planes are obtained.

**Figure 2:**
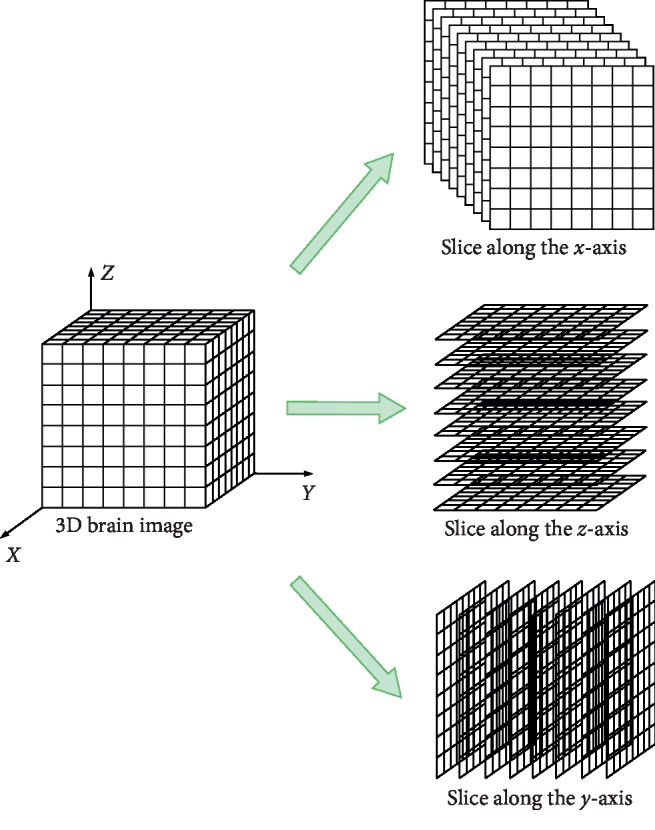
The process of transforming a 3D brain image into three multichannel 2D images.

Based on the idea of channels in CNN, regard each image on the coronal plane as an image in a channel and transform this group of images into a 2D image with dim*X* channels. Similarly, a 2D image with dim*Y* channels and a 2D image with dim*Z* channels can be obtained. Thus, three multichannel 2D images are transformed from each 3D brain image, as shown in Figure 2. These three channels, importantly, also match the stereotaxic brain space, a reference tradition used in MRI research [14]: *x* (sagittal), *y* (coronal), and *z* (axial). The three multichannel 2D images are then input into three parts of 2D CNN.

#### 2.1.2. Convolutional Layer

Each 2D CNN of M2D CNN processes the convolution computing for the input multichannel 2D image and extracts features on its plane. Each convolutional kernel is convolved across the width and height of 2D input volumes from previous layer, computing the dot product between the kernel and the input. The results of all input volumes are summed to arrive at the result of this kernel, producing a 2-dimensional activation map for each kernel.

#### 2.1.3. Pooling Layer

Each convolutional layer is followed by a max pooling layer, which performs feature selection and filtering on the result of convolutional layer to achieve smooth compression in a degree.

#### 2.1.4. Merge Layer

The outputs of three parts of the 2D CNN architecture are concatenated into a 1D vector, which represents the combined spatial features learned from three orthogonal planes of the 3D brain image.

#### 2.1.5. Fully Connected Layer

The concatenated vector from the merge layer is input into the fully connected layer, where the units are fully connected to the units in previous layer. Feature combination is performed and complex nonlinear relationships are modeled in this layer. Due to the limited number of samples of fMRI data, a few fully connected layers are enough. In our experiment, we use one hidden layer.

#### 2.1.6. Output Layer

The model outputs several real numbers, representing the probability of each classification category that the sample may belong to. The softmax function, as shown in equation (1), is used for probability calculation of each category:

(1) $$\begin{array}{c} \sigma {\left(z\right)}_{j}=\frac{e^{z_{j}}}{\sum_{k=1}^{K}e^{z_{k}}}. \end{array}$$

### 2.2. Comparison Models

Given the above M2D CNN model, we use the following models as benchmarks for comparison of classification performance when given the same input data and output categories.

#### 2.2.1. PCA + SVM

With the principal component analysis- (PCA-) based SVM model [8, 15], all voxel features of 3D brain images are flattened into a 1D vector. After dimensionality reduction by PCA, the vector is input into SVM for training and classification.

#### 2.2.2. mv2D CNN

**Figure 3:**
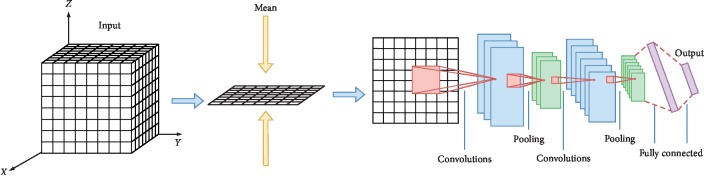
Architecture of mv2D CNN.

We discuss here the mean-value 2D CNN for analysis of voxel-wise fMRI brain images [4]. 3D brain images are converted to 2D mean-value images on the axial plane by computing the mean value of voxel features along the *z*-axis (that is, compressing the third dimension by averaging the values). The 2D mean-value images are then input into 2D CNN, as shown in Figure 3.

#### 2.2.3. 1D CNN

**Figure 4:**
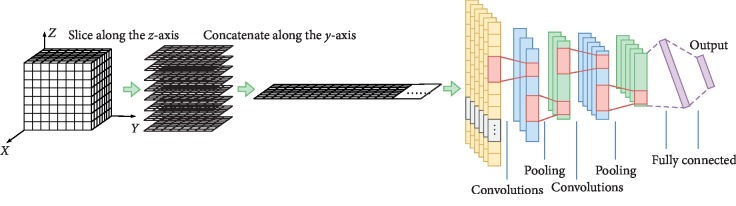
Architecture of 1D CNN.

To compare the different convolution methods, we provide a baseline and design an 1D CNN for analysis of voxel-wise fMRI data. In this 1D CNN model, each 3D brain image is converted to vector sequence. In our experiment, this is done by slicing along the *z*-axis to get a number of 2D images on the axial plane and concatenating them in order along the *y*-axis to obtain a vector sequence. The vector sequences from 3D brain images are input into 1D CNN, as shown in Figure 4.

#### 2.2.4. 3D CNN

**Figure 5:**
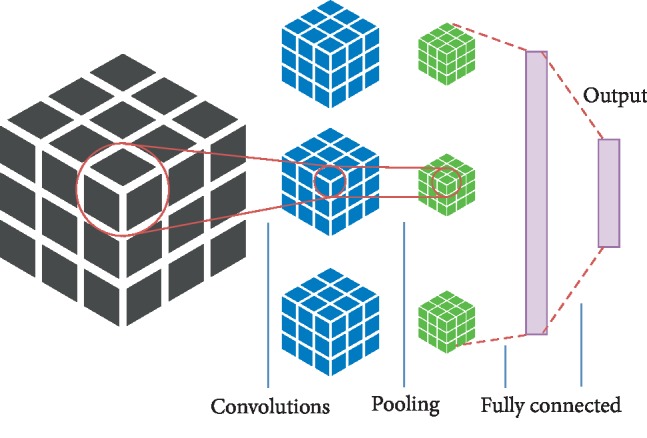
Architecture of 3D CNN.

This is the typical 3D CNN for the analysis of voxel-wise fMRI data, as done in previous work [7, 8]. Input of 3D brain images is sent into the 3D CNN, as shown in Figure 5.

#### 2.2.5. 3D SepConv

Separable convolutions could be intuitively understood as a way to factorize a convolution kernel into two smaller kernels. In this study, 3D SepConv model uses the same structure as the above 3D CNN model but the 3D convolutional layer is changed to 3D separable convolution layer.

#### 2.2.6. s2D CNN

**Figure 6:**
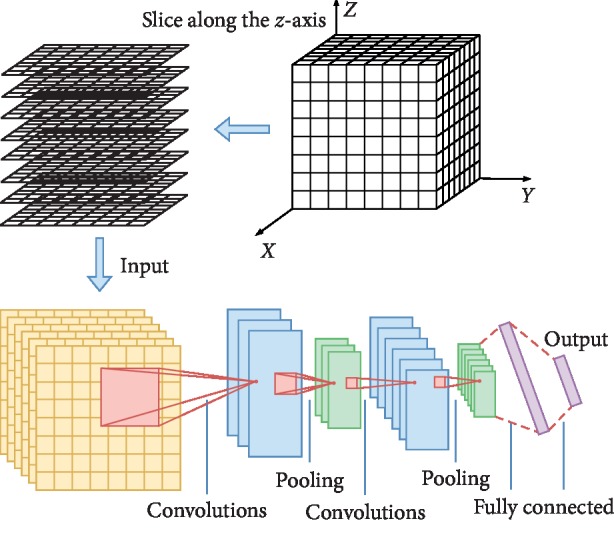
Architecture of s2D CNN.

The single-channel 2D CNN is designed in this study for analysis of voxel-wise fMRI data. The difference between this model and the M2D CNN is that s2D CNN only focuses on one dimension (axial, the *z*-axis). In our experiment, this means that, for a 3D brain image, slice along the *z*-axis in per unit length to get a number of images on the axial plane and transform them into a multichannel 2D image, inputting into 2D CNN, as shown in Figure 6.

#### 2.2.7. M2D CNN

The proposed multichannel 2D CNN is described in Section 2.1. Transform each 3D brain image into three multichannel 2D images by the method described in detail in Section 2.1 and input them into M2D CNN (see Figure 1).

## 3. Experiments

### 3.1. Experimental Dataset and Preprocessing

The fMRI data from the motor task of the public WU-Minn Human Connectome Project (HCP) were used as the benchmark data for our experimental comparison between different models (http://protocols.humanconnectome.org/HCP/3T/task-fMRI-protocol-details.html). During the motor task, the MRI continuously scans the participant's brain, during which time the participants were presented with visual cues/labels that ask them to perform specific actions, including taping their left fingers (with label “Left Hand”) or right fingers (with label “Right Hand”) or squeezing their left toes (with label “Left Foot”) or right toes (with label “Right Foot”) or moving their tongue (with label “Tongue”). These 5 types of movement were performed twice in the whole motor task (with a total of 10 movement blocks, each corresponding to one movement). Furthermore, there were three 15-second fixation blocks in the motor task, intermixed with the movement blocks. For more details of fMRI acquisition and preprocessing, see [16, 17]. By extracting the fMRI data from each movement block for analysis, our deep learning methods presented in this study aim at classifying participants' movement types for identifying the relationship between human behavior and brain activity.

After filtering the subjects that had anomalous data or had no motor task data, we obtained a total of 995 subjects, with each subject containing 4D fMRI data with the size of 91 × 109 × 91 × 284. This means that there are 284 time-series frames of 3D brain images with the size of 91 × 109 × 91 (voxel size = 2 × 2 × 2 mm^3^) scanned during the motor task. According to the flow scheme of motor task, each subject underwent 3 fixation blocks and 10 movement blocks, and the fixation blocks were used as the baseline to compute the BOLD values of each voxel. In our experiments, we used the BOLD signals in the movement blocks to generate samples of 3D brain images for training and classification as follows.

For 3D images in fixation blocks, we computed the mean-value 3D images as the BOLD baseline value for each voxel. For 3D images in movement blocks, the widely used method of mean percent signal change (Mean PSC) is applied, which computes the average change for every voxel relative to the baseline value, thereby transforming into a frame of mean 3D brain image. Mean PSC for every voxel is computed as

(2) $$\begin{array}{c} p=\frac{\sum_{i=1}^{N}y_{i}}{\bar{y}\cdot N}\cdot 100. \end{array}$$

In equation (2), *N* represents the number of 3D brain images in this movement block and *y*_*i*_ represents the voxel value in the *i*th images. $\bar{y}$ represents the baseline value of the voxel computed from fixation block. *p* is the average change of the voxel. The average changes for all voxels are remodeled into a mean 3D image, therefore converting the data in a movement block to a 3D brain image. For every subject, 10 samples of 3D brain image are obtained from the subject's 10 movement blocks.

Data normalization is achieved by subtracting the mean and dividing the standard deviation on every voxel for all samples. Then, the movement type of each sample is used as its classification label. There are a total of 9,950 samples of 3D brain images with the size of 91 × 109 × 91 from the 995 subjects.

### 3.2. Model Parameters

The comparison models and the corresponding parameters are configured as follows.

#### 3.2.1. PCA + SVM

Each 3D image has 902,629 dimensions after being flattened. We test on the data subset of 200 subjects from all subjects for PCA, and the variance percentage of the original vector (the percentage of preserved information) reaches 85% [8] when the number of chosen principal components is 500. Considering the limitation of memory, the dimension of the resulting vector of PCA is set to 500. SVM model sets the value of parameter C to 1.0 and uses the L2-regularization and linear kernel.

#### 3.2.2. M2D CNN

The M2D CNN includes 3 parts of 2D CNN architecture, each containing 2 convolution layers and 2 max pooling layers, as described in Section 2.1 in detail.

#### 3.2.3. 1D CNN, s2D CNN, mv2D CNN, and 3D CNN

These CNN models contain 2 convolution layers, 2 max pooling layers, a fully connected layer, and an output layer. The 2 convolution layers of CNN above contain 16 and 32 kernels, respectively, with the kernel length of 3. Each convolution layer is followed by a max pooling layer with the window length of 2 and the batch normalization. Each fully connected layer contains 128 nodes, with the dropout function randomly dropping 50% input units to prevent overfitting.

#### 3.2.4. 3D SepConv

3D SepConv model uses the same structure and parameters as 3D CNN model except that the second 3D convolutional layer is changed to the separable convolution layer. The separate convolution splits a kernel into two smaller kernels that do the depth-wise convolution and the pointwise convolution. Each input channel is spatially convolved separately by the depth-wise convolution; then the resulting outputs are mixed via pointwise convolutions with a kernel size of 1 × 1 × 1. The 3D separate convolution module is implemented according to the code of Spasov et al [12].

All the deep learning models above use softmax function in the output layer and LeakyReLU function in the other layers as activation function [18]. The Adam Optimizer with initial learning rate of 0.0025, reduced by half every 50 epochs, is used for model training, and categorical cross-entropy is selected as loss function. The training model after each epoch is validated on validation data and the one with best validation result is saved. The model stops training when the training loss has stopped decreasing for six epochs. Fivefold cross-validation is performed for the classification of the 5 movement types in the motor task. In each fold, 80% of the samples are used as training data (in which 10% is selected as validation data) and the remaining 20% are used as testing data. Samples are split according to subjects to make sure that every subject's 10 samples are from the same part of data.

We used for our experiments a specialized computer with i7-6700K CPU, 64 GB RAM, and a NVIDIA GTX 1080 Ti Graphics Processing Unit (GPU). The models are coded with Python. Keras (https://keras.io) and TensorFlow (https://www.tensorflow.org) as backend are also applied to implement the model. In the spirit of open science and to promote reproducibility [19], we make our source codes publicly available on our project website (https://github.com/largeapp/M2DCNN).

### 3.3. Results

#### 3.3.1. Classification Performance on Large Dataset

**Figure 7:**
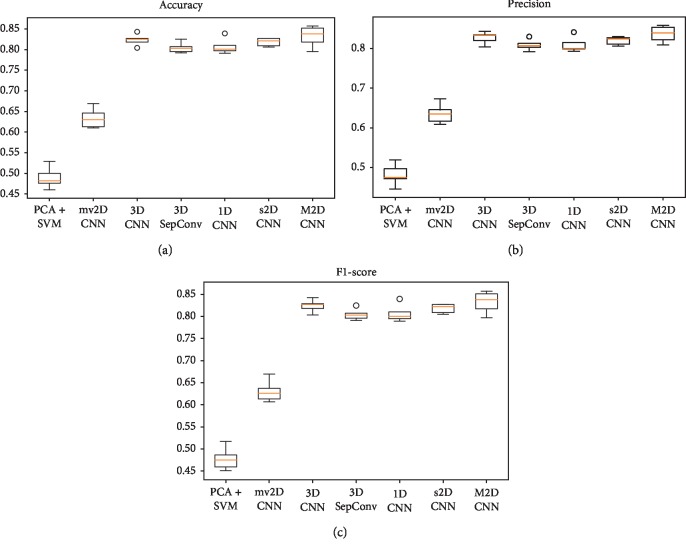
Box plots for accuracy, precision, and F1-score for classification task on 995 subjects of different learning models over 5-fold cross-validation. The middle line in each box represents the median value. The circle represents the outlier.

Table 1 and Figure 7 show the experimental results by comparing the accuracy, precision, and F1-score rates over 5-fold cross-validation for the 9950 samples' data from all 995 subjects. The accuracy of the M2D CNN, 3D CNN, 3D SepConv, s2D CNN, and 1D CNN models all reached above 80%, which is much better than the accuracy of the PCA + SVM and mv2D CNN models. This may be because several CNN models have preserved all voxel features of the 3D brain images. Most notably, the M2D CNN model proposed in this study reached an accuracy of 83.2% in classification, with a precision rate of 83.6%. Thus, M2D CNN outperforms all other models, such that M2D CNN > 3D CNN > s2D CNN > 1D CNN > 3D SepConv.

#### 3.3.2. Impacts of Sample Size

To further verify the effect of sample size, we randomly selected 200 and 500 subjects out of the 995 subjects, respectively, to form the datasets with 2000 and 5000 samples. The deep learning models in comparison were all tested on the three datasets (i.e., from 200, 500, and 995 subjects).

Table 2 presents the classification accuracy over different sample size of data. As data size grows, the accuracy of each model increases. The accuracy of 3D CNN, 3D SepConv, and M2D CNN is better than the other CNN models for 200 subjects. The accuracy of M2D CNN is better than the other CNN models for 500 subjects.

#### 3.3.3. Number of Parameters and Training Time

Table 3 shows the number of units connected to the fully connected layer and the total number of parameters of each CNN model based on the experimental configuration in Section 3.2. The fully connected layer leads to a sharp increase in the number of parameters.

3D CNN uses kernels convoluting in 3 dimensions and produces 352,800 units after flattening the result from convolution and pooling layers. 3D SepConv reduces the parameters by using separate convolution in comparison to general 3D CNN, but there are still many parameters since the large number of parameters resides at the fully connected networks in this model. In contrast, the 2D CNN models convoluting on one plane such as the mv2D CNN and s2D CNN models contain only 16,800 units. M2D CNN uses 2D convolution kernels, which reduces the number of units from convolution to the fully connected layer, and its concatenated vector from 3 parts of convolution on each plane contains 47,712 units, leading to a total of 6,355,717 parameters, which are much fewer than 45,174,181 for 3D CNN.

Table 3 also shows the total training time and total epochs over 5-fold cross-validation for all CNN models with 2000 samples from 200 subjects. We have noticed that the training time would be affected by the underlying software (such as Keras, TensorFlow, and operating system) and hardware systems (GPU device and CPU host), especially when the large data need to be read from the hard disk during the model training. To reduce the impact of overhead of reading data from hard disk, we selected smaller data (2000 samples) and loaded all these data to memory of the host computer before training. So we could get the approximate training time under the specific computer in this experiment. From Table 3, it can be seen that 3D CNN, 3D SepConv, and M2D CNN models use more time to train than the 1D and 2D CNN models. 3D SepConv model uses the same input data and has fewer parameters than 3D CNN model, but 3D SepConv model uses more training time in our experiments; the reason may be the fact that the implementation of the 3D SepConv model is not efficient. M2D CNN model has less training time than 3D CNN and 3D SepConv models.

#### 3.3.4. Overfitting

**Figure 8:**
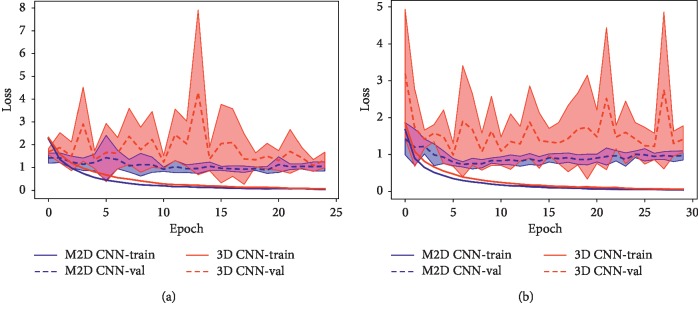
Mean training loss (in solid lines) and mean validation loss (in dashed lines) for 3D CNN (red) and M2D CNN (blue). The standard deviation is indicated by the shadow area in the image. (a) 2000 samples. (b) 5000 samples.

Figure 8 shows the standard deviation of the mean training and validation losses across the 5-fold training and validation for M2D CNN model and 3D CNN model, respectively, with 2000 samples from 200 subjects (see Figure 8(a)) and 5000 samples from 500 subjects (see Figure 8(b)). It could be seen that the training losses of 3D CNN model decrease quickly but the validation losses fluctuate sharply. In contrast, both the training and validation losses decrease steadily for the M2D CNN model, which indicates that the data overfitting problem is alleviated in M2D CNN.

In summary, the proposed M2D CNN is more cost-effective for the classification of task-evoked fMRI than the other comparison models tested in this experiment. M2D CNN outperforms the other models with the best classification performance and achieves higher parameter-efficiency than 3D CNN with fewer parameters, thus alleviating data overfitting.

## 4. Conclusion

Due to the complex spatiotemporal structure of fMRI data and the large amount of voxel features, the existing deep learning methods need to take into account the integrity of the 3D or 4D information of the whole brain contained in fMRI data, along with considerations of the feature extraction capacity and training efficiency of the relevant models. This paper proposes a novel M2D CNN model, using three multichannel 2D CNN networks in parallel to preserve voxel features of 3D brain images and integrating three-dimensional information by fully connected neural network. The results of our experiments show that M2D CNN outperforms the other comparison models and achieves the best classification performance.

There are two limitations in the work presented here: (i) the dataset is limited to the motor task—it would be important to see whether the proposed M2D CNN model excels in classifying fMRI data based on cognitive tasks; (ii) the dataset is limited in the total sample size—9,950 samples of 3D brain images from all 995 subjects remain to be of small scale as compared with the computational power of deep learning models. Future work should focus on the performance of our proposed model for other large-scale resting-state as well as task-evoked fMRI data based on cognitive tasks of memory, language, and vision and perception.

## Figures and Tables

**Table 1:** Classification results for all deep learning models on data of 995 subjects (mean ± std).

Model	Accuracy^*∗*^ (%)	Precision (%)	F1-score
PCA + SVM	48.94 ± 2.36	48.17 ± 2.48	0.4779 ± 0.0232
mv2D CNN	63.36 ± 2.19	63.59 ± 2.27	0.6306 ± 0.0222
3D CNN	82.34 ± 1.27	82.68 ± 1.39	0.8239 ± 0.0130
3D SepConv	80.44 ± 1.16	80.88 ± 1.24	0.8043 ± 0.0116
1D CNN	80.76 ± 1.69	80.94 ± 1.73	0.8068 ± 0.0178
s2D CNN	81.80 ± 0.89	81.95 ± 0.97	0.8179 ± 0.0094
M2D CNN	**83.20** ± **2.29**	**83.63** ± **1.87**	**0.8321** ± **0.0223**

^*∗*^Note: accuracy by chance is 20% (i.e., given 5 types of movement behavior).

**Table 2:** Classification accuracy over different sample sizes.

Model	Accuracy (mean ± std) over different sample sizes		
	2000 samples (200 subjects) (%)	5000 samples (500 subjects) (%)	9950 samples (995 subjects) (%)
mv2D CNN	53.70 ± 4.20	60.88 ± 2.28	63.36 ± 2.19
3D CNN	72.70 ± 2.54	77.36 ± 1.95	82.34 ± 1.27
3D SepConv	73.60 ± 1.77	77.24 ± 2.79	80.44 ± 1.16
1D CNN	67.40 ± 2.92	76.52 ± 1.09	80.76 ± 1.69
s2D CNN	66.20 ± 3.19	76.64 ± 1.96	81.80 ± 0.89
M2D CNN	71.70 ± 1.81	79.44 ± 1.70	83.20 ± 2.29

**Table 3:** A comparison of model units, parameters, and training time.

Model	Unit number input to fully connected layer	Total number of parameters in models	Training time (S) (mean ± std)	Total number of epochs (mean ± std)
mv2D CNN	16,800	2,223,877	909 ± 134	54 ± 8
3D CNN	352,800	45,174,181	1156 ± 185	39 ± 6
3D SepConv	352,800	45,161,301	1601 ± 196	41 ± 5
1D CNN	79,296	10,474,501	834 ± 157	39 ± 7
s2D CNN	16,800	2,236,837	565 ± 102	31 ± 6
M2D CNN	47,712	6,355,717	1074 ± 348	39 ± 13

## Data Availability

The data used to support the findings of this study are available from the corresponding author upon request. The task-based fMRI dataset analyzed during this study is available in the Human Connectome Project repository (http://www.humanconnectome.org/). The codes used for the reported experiments are available at https://github.com/largeapp/M2DCNN.

## References

[1] Mitchell T. M., Shinkareva S. V., Carlson A. (2008). Predicting human brain activity associated with the meanings of nouns. *Science*.

[2] Kriegeskorte N. (2015). Deep neural networks: a new framework for modeling biological vision and brain information processing. *Annual Review of Vision Science*.

[3] LeCun Y., Bengio Y., Hinton G. (2015). Deep learning. *Nature*.

[4] Nathawani D., Sharma T., Yang Y. (2016). *Neuroscience Meets Deep Learning*.

[5] Hon M., Khan N. M. Towards Alzheimer’s disease classification through transfer learning.

[6] Zafar R., Kamel N., Naufal M. (2017). Decoding of visual activity patterns from fMRI responses using multivariate pattern analyses and convolutional neural network. *Journal of Integrative Neuroscience*.

[7] Li H., Satterthwaite T. D., Fan Y. Brain age prediction based on resting-state functional connectivity patterns using convolutional neural networks.

[8] Li X., Dvornek N. C., Papademetris X. 2-channel convolutional 3D deep neural network (2CC3D) for fMRI analysis: ASD classification and feature learning.

[9] Wang X., Liang X., Zhou Y. (2018). Task state decoding and mapping of individual four-dimensional fMRI time series using deep neural network. http://arxiv.org/abs/1801.09858.

[10] Sarraf S., Tofighi G. (2016). Classification of Alzheimer’s disease using fMRI data and deep learning convolutional neural networks. http://arxiv.org/abs/1603.08631.

[11] Chollet F. Xception: deep learning with depthwise separable convolutions.

[12] Spasov S., Passamonti L., Duggento A., Liò P., Toschi N. (2019). A parameter-efficient deep learning approach to predict conversion from mild cognitive impairment to Alzheimer’s disease. *Neuroimage*.

[13] Simonyan K., Zisserman A. (2014). Very deep convolutional networks for large-scale image recognition. http://arxiv.org/abs/1409.1556.

[14] Huettel S. A., Song A. W., McCarthy G. (2014). *Functional Magnetic Resonance Imaging*.

[15] Hazlett H. C., Gu H., Munsell B. C. (2017). Early brain development in infants at high risk for autism spectrum disorder. *Nature*.

[16] (2017). Human Connectome Project. WU-Minn HCP 1200 subjects data release reference manual. https://www.humanconnectome.org/storage/app/media/documentation/s1200/HCP_S1200_Release_Reference_Manual.pdf.

[17] Glasser M. F., Sotiropoulos S. N., Wilson J. A. (2013). The minimal preprocessing pipelines for the Human Connectome Project. *Neuroimage*.

[18] Maas A. L., Hannum A. Y., Ng A. Y. Rectified nonlinearities improve neural network acoustic models.

[19] Munafò M. R., Nosek B. A., Bishop D. V. M. (2017). A manifesto for reproducible science. *Nature Human Behaviour*.

